# *DSN1* may predict poor prognosis of lower-grade glioma patients and be a potential target for immunotherapy

**DOI:** 10.1080/15384047.2024.2425134

**Published:** 2024-11-18

**Authors:** Yulong Jia, Meiling Liu, Han Liu, Wenjia Liang, Qingyun Zhu, Chao Wang, Yake Chen, Yanzheng Gao, Zhendong Liu, Xingbo Cheng

**Affiliations:** a Department of Neurosurgery, Henan Provincial People’s Hospital, People’s Hospital of Zhengzhou University, School of Clinical Medicine, Henan University, Zhengzhou, China; b School of Clinical Medicine, Sanquan College of Xinxiang Medical University, Xinxiang, Henan, China; c Department of Clinical Medicine, Medical College of Jinzhou Medical University. Taihe District, Jinzhou, Liaoning Province, China; d Henan Provincial People’s Hospital, People’s Hospital of Henan University, Zhengzhou, Henan Province, China; e Department of Neurobiology, School of Basic Medical Sciences, Harbin Medical University, Harbin, Heilongjiang, P. R. China; f School of Pharmacy, Xinxiang Medical University, Xinxiang, Henan, China; g Department of Surgery of Spine and Spinal Cord, Henan Provincial People’s Hospital, Henan Province Intelligent orthopedic technology innovation and transformation International Joint Laboratory, Henan Key Laboratory for intelligent precision orthopedics, People’s Hospital of Zhengzhou University, People’s Hospital of Henan University, Zhengzhou, Henan, China

**Keywords:** Low-grade glioma, *DSN1*, biomarker, prognosis, immunotherapy

## Abstract

DSN1 has been previously found to be positively correlated with various cancers. However, the effect of DSN1 or its methylation on the prognosis, molecular characteristics, and immune cell infiltration of low-grade glioma (LGG) has not yet been studied. We obtained 1046 LGG samples from the The Cancer Genome Atlas, The Chinese Glioma Genome Atlas (CGGA) microarray, and CGGA RNA-Seq databases. Bioinformatic methods (gene set enrichment analysis (GSEA), chi-square test, multivariate), and laboratory validation were used to investigate DSN1 in LGG. The expression levels of DSN1 mRNA and protein in LGG were substantially higher than those in normal brain tissue, and their expression was negatively regulated by methylation. The survival time of patients with low expression of DSN1 and cg12601032 hypermethylation was considerably prolonged. DSN1 was a risk factor, and of good diagnostic and prognostic value for LGG. Importantly, the expression of DSN1 is related to many types of tumor-infiltrating immune cells and has a positive correlation with PDL1. DSN1 promoted the activation of multiple cancer-related pathways, such as the cell cycle. Additionally, knockdown of DSN1 substantially inhibited the proliferation and invasion of LGG cells. To the best of our knowledge, this study is the first comprehensive analysis of the mechanism of DSN1 leading to poor prognosis of LGG, which provides a new perspective for revealing the pathogenesis of LGG. DSN1 or its methylation has diagnostic value for the prognosis of glioma, and may become a new biological target of anti-tumor immunotherapy.

## Introduction

Low-grade glioma (LGG) is thought to arise from glial precursor cells and is one of the most common primary malignant tumors in the brain, accounting for 17–22% of all primary brain tumors.^1^ LGG has a certain degree of inertia; however, adult patients with LGGs who are not treated in time will eventually develop high-grade gliomas. The malignant development of LGG endangers patient health and increases the national medical burden.^2^ Currently, the internationally recognized LGG treatment is a combination of surgery and chemotherapy.^3^ However, owing to tumor-invasive growth and the influence of the blood-brain barrier, the survival time of patients with LGG remains unsatisfactory.^4–6^ Therefore, an increasing number of researchers have recently turned their attention to biomarkers of tumor genomic instability to diagnose and improve the prognosis of patients with LGG.^7–9^ Presently, some biological treatment targets such as *PDL1*, *MGMT*, *STAT3*, *EGFR*, and aldehyde dehydrogenases are widely used in the prognostic evaluation and diagnosis of glioma.^4,10–12^ However, LGG is a polygenic genetic disease that is regulated by many factors. Therefore, it is necessary to identify additional biological targets to improve the prognosis and treatment of patients with gliomas.

In addition to the common characteristic of genomic instability, the roles of the immune microenvironment and checkpoints in LGGs have been recognized. The infiltration level of immune cells, and the inhibition of immune checkpoints are specific mechanisms of tumor immune escape.^13,14^ Therefore, numerous studies have developed new treatments for immunotherapy, with *PD1* and *PDL1* being the best-known targets.^15^ Although inhibitors blocking *PD1/PDL1* can inhibit tumorigenesis, only a subset of patients benefit from the clinical responses; the treatment of most patients did not achieve notable therapeutic effects, and resulted in some toxic and side effects.^16^ the complex regulatory mechanism of the tumor immune microenvironment has not yet been elucidated, therefore, it is difficult to improve the clinical effect of antitumor immunotherapy. Exploring the key regulatory molecules formed in the special immunosuppressive tumor microenvironment is important for improving the understanding of tumor pathological mechanisms and providing a potential target to establish individualized multi-target anti-tumor immunotherapy, which has important clinical significance.

The development of cross-disciplines leads to the revelation of the regulatory mechanisms of tumor immunity. Recently, it has been established that an abundance of immune checkpoints is related to the cell cycle. CDK4/6, an essential molecule for the cell cycle, has been reported to stabilize the expression of Cullin 3-SPOP E3 ligase by regulating the phosphorylation of speckle-type POZ protein (SPOP), which mediates the proteasomal degradation of PDL1.^17^ Thus, targeting cell cycle-related factors could further explore the mechanisms of tumor immunity. Dysregulation of the cell cycle pathway, which is a central mechanism regulating cell proliferation, has been identified to be involved in cancer pathogenesis.^18^ It has been reported that *DSN1* depletion blocks the cell cycle at the G2/M phase, and inhibits the invasion and migration of colorectal cancer cells.^19^ Additionally, *DSN1* has been widely reported in various cancers, including hepatocellular carcinoma, gastric cancer, and osteosarcoma.^20–22^ Interestingly, *DSN1* has also been shown to promote immune cell infiltration in multiple cancers.^23^ However, the potential value of *DSN1* expression in the prognostic evaluation of patients with LGG has not yet been reported.

Therefore, this study used three datasets from the The Cancer Genome Atlas (TCGA), The Chinese Glioma Genome Atlas (CGGA) microarray, and CGGA RNA-Seq databases to reveal the potential relationship between the expression of *DSN1* and patient survival in terms of both molecular and clinical aspects. Gene set enrichment analysis (GSEA) was performed to determine the biological functions of *DSN1*. Laboratory experiments were performed to explore the effects of *DSN1* on the proliferative and invasive abilities of the LGG cells. To our knowledge, this study is the first to investigate the relationship between *DSN1* and the prognosis of patients with LGG using multiple databases.

## Materials and methods

### Public data collection

TCGA is the current mainstream public data platform that has milestone significance in cancer genomics projects^24^ (https://cancergenome.nih.gov/). It contains clinical data for various human cancers, sample information, and a complete range of data, which makes it convenient for researchers to analyze key genes involved in tumor regulation. In this study, the transcriptome data of 503 LGGs were included from the TCGA database after removing some samples with missing clinical information, such as age and survival status (Table S1). We also obtained 501 methylation data points and corresponding clinical information from the UCSC Xena database (https://xenabrowser.net/).^25^ These databases can be used for biometric analyses to reveal the molecular mechanisms of DSN1 in patients with LGG.

CGGA (http://www.cgga.org.cn/), established by the Beijing Tian Tan Hospital and Peking University, is an authoritative public database focusing on gliomas.^26^ The database contains a variety of high-throughput data and the corresponding clinical information libraries. After excluding data with incomplete clinical information, such as missing age and survival, this study obtained a CGGA RNA-Seq set of 403 LGG samples and CGGA mRNA-array data containing 140 LGG samples. Multiple databases were used to verify the relationship between *DSN1* and LGG.

The Gene Expression Omnibus (GEO) database has a large amount of gene sequencing data.^27^ In addition, the database contains the survival time and status of many patients with LGG (https://www.ncbi.nlm.nih.gov/geo/). After a series of screenings, the study included three datasets in the meta-analysis: GSE43378 (*n* = 18), GSE4412 (*n* = 26), and GSE50025 (*n* = 34).

### Meta-analysis

To further reveal the relationship between *DSN1* expression and LGG prognosis, we searched the expression profiles of *DSN1* and LGGs using multiple authoritative public databases and retrieval platforms. We found no studies reporting an association between *DSN1* and LGG prognosis. Therefore, we included six datasets (TCGA RNA-seq (503), CGGA-mRNA-array (140), CGGA-RNA-seq (403), GSE43378 (*n* = 18), GSE4412 (*n* = 26), and GSE50025 (*n* = 34)) in our meta-analysis. After we obtained six independent datasets from different sources, we conducted Cox analysis individually to obtain the HR value of a single dataset, and then each HR value obtained from the six different datasets was subjected to mate analysis. The heterogeneity of the six datasets was assessed using the Q-test, and I^2^ = 90 was found to be > 50%. We chose to use a random-effects model for data analysis because of the large differences between the datasets.

### Gene expression levels

GEPIA (http://gepia.cancer-pku.cn/) is a database of human tumor samples established by Peking University.^28^ It is also a free online platform that allows researchers to study the expression levels of target genes in different tumors anytime and anywhere. Additionally, the platform can be used to explore the differences in gene expression between specific tumors and corresponding normal groups. Therefore, we used the GEPIA database to explore the level of *DSN1* expression in LGG and normal tissues. An expression difference map was obtained for tumor tissues (*n* = 518) and normal control tissues (*n* = 207). HPA database is a professional data platform focusing on tumor research, which provides protein expression data of many common human tumors and allows researchers to obtain original immunohistochemical expression images in tumors and present and publish them in articles (https://www.proteinatlas.org).^29^ Therefore, we obtained immunohistochemical images of *DSN1* in LGG and normal brain tissues from the HPA database to reveal changes in the protein expression level of *DSN1* in LGG.

### Immune correlation analysis of DSN1 in LGG

Based on the impact of the tumor immune microenvironment on the prognosis of tumor patients and the rise in antitumor immunotherapy,^30^ researchers have attempted to identify new biological targets for antitumor therapy to improve the prognosis of tumor patients. Therefore, this study also attempted to reveal the regulatory effect of *DSN1* on the tumor immune microenvironment in LGG. The TIMER is an online analysis tool for tumor immune microenvironment research (https://cistrome.shinyapps.io/timer/) that provides the expression relationship between the target gene and immune cells that infiltrate the tumor microenvironment.^31^ To reveal the relationship between the expression level of *DSN1* and the degree of immune cell infiltration in the LGG microenvironment, we input the gene symbol of *DSN1* into the module of “gene” in TIMER to determine the influence of *DSN1* on the infiltration level of six different immune cells. Subsequently, the module of “survival” in TIMER was used to revealed the effects of *DSN1* and six different immune cells on the prognosis of LGG patients. The Kaplan-Meier method can also be used to draw survival maps of immune infiltration and genes to visualize survival differences, and the p-value of the log-rank test for the survival curves of the two groups can also be generated in each graph. The module of “SCNA” in TIMER was used to analyze the comparison of tumor invasion levels between tumors with different somatic cell copy number changes in *DSN1*. Copy number variations, including deep deletion, arm-level deletion, diploid/normal, arm-level gain, and high amplification and the box plot reveals the distribution of immune subsets in each copy number state in LGG. The infiltration level for each SCNA category was compared with the normal level using a two-sided Wilcoxon rank-sum test. In addition, the module of “mutation module” in TIMER was used to analyze the effect of *IDH* mutation on the level of immune cell infiltration. Finally, this study draws a scatter plot of the expression of *DSN1* and well-known immune checkpoints (*PDCD1* (PD1), *CD274* (PDL1), *PDCD1LG2* (PDL2) and *CTLA4* (CD152)) in the “correlation” module with spearman’s rank correlation coefficient. P-values <0.05, were considered significant in all the above analysis results.

### Gene set enrichment analysis

GSEA is the current mainstream bioinformatics analysis method that provides simple and clear association analysis between several gene sets and the entire transcriptome.^32^ To further understand the biological mechanism and indirectly interpret the function of *DSN1*, GSEA (version 4.0.3) was performed. The mRNA data downloaded from TCGA and CGGA databases were batch-corrected and normalized using the Limma software package. Based on the expression level of *DSN1*, the data were divided into high- and low-expression groups. The number of permutations was set to 1000, the “KEGG signal pathway” in the gene database was selected, and the *p*- and q-values were screened to obtain some highly correlated signal pathways.

### Cell culture and clinical sample collection

The LGG cell line, SHG-44, was purchased from BlueBio (Beijing, China). SHG-44 cells were cultured in complete medium containing 10% fetal bovine serum (FBS), and 1% penicillin and streptomycin, and placed in a cell incubator at 37°C with 5% CO_2_. For subsequent experiments, cell passage was performed at nearly 100% cell fusion, and the complete medium was replaced as required. SHG-44 cells were treated with the three siRNAs purchased from GenePharma (Shanghai, China). An siRNA negative control (siNC) was used as the control group. The second siRNA (si2), which had the highest knockdown efficiency among the three interference sequences targeting *DSN1*, was used for subsequent cell experiments with the following sequences: Sense: 5’- GCGAGCAAGUAUGAAAGAATT −3’ and Anti-sense: 5’- UUCUUUCAUACUUGCUCGCTT −3’. The knockdown of si2, which targets DSN1 at the protein level, was also detected.

LGG brain tissue samples were collected from patients with clinically diagnosed grade II-III gliomas, and normal brain tissues were collected from patients with clinically diagnosed epilepsy in Henan People’s Hospital. First, 5 cases of normal brain tissues and 10 cases of LGG samples from the laboratory were collected to validate the transcriptomic level of DSN1. Secondly, additional LGG samples were used for immunohistochemistry. All brain tissue samples were collected during surgery and preserved in a 4% polyformaldehyde solution at room temperature. All participants from Henan People’s Hospital signed informed consent forms in advance, and the study was approved by the Ethics Committee of Henan People’s Hospital (2020107).

### Real-time quantitative PCR

Total RNA was extracted from SHG-44 cells treated with siRNA for 72 h, and then reverse-transcribed to cDNA. RT-qPCR was performed to detect mRNA levels of *DSN1* in all samples. The specific primer sequences for *DSN1* was: Forward 5′- AATCTCTTCGGCGTCGTACC −3′; Reverse 5′- TGCCCTCGGGTTTCATTTCA −3′. The specific primer sequence of 18S, which was an internal parameter, was: Forward 5′-GTAACCCGTTGAACCCCATT-3′, and Reverse 5′-CCATCCAATCGGTAGTAGCG-3′.

### Immunohistochemistry

To identify the protein levels of *DSN1* at the tissue level, three LGG and normal brain tissue samples were selected for immunohistochemistry. Samples were first dewaxed, dehydrated, and incubated with EDTA antigen repair solution and endogenous peroxidase blockage. After sealing with a 10% BSA solution, the tissue sections were incubated with a specific antibody targeting *DSN1* at 4°C overnight. The protein of *DSN1* was stained using the DAB method after incubation with a secondary antibody conjugated with HRP. The nuclei were stained with a hematoxylin solution. Finally, the images were processed and quantified using ImagePro-Plus software.

### CCK8 assay and colony formation assay

SHG-44 cells treated with siRNA were inoculated into a 96-well plate at a density of 2,000 cells per well. It was set to 0 h when the cells attached to the plate. At this point, the working solution of cell-counting-kit-8 (CCK8) was added to the 96-well plate and cultured in the cell incubator at 37°C for 4 h. The absorbance of the cell culture medium at 450 nm was measured by a Microplate Reader. Subsequently, the absorbance values of treated cells at 24 h, 48 h and 72 h were also measured using the same method.

SHG-44 cells were seeded into 6-well plates at a density of 500 cells/well and cultured in complete medium at 37°C for 14 d. After colony formation, the cells were fixed at room temperature with 4% polyformaldehyde for 30 min, and then stained with crystal violet solution. The number of colonies was counted and statistically analyzed.

### Wound healing and transwell assays

SHG-44 cells transfected with siRNA were cultured until 100% fusion was achieved, and then a sterile pipette tip was used to create a wound. After cleaning with phosphate buffered saline (PBS), the maintenance medium (without FBS) was added to the 6-well plates. The relative distance between cells on both sides of the same wound were photographed at the set time point of 0 h and 24 h.

Medium with 5% FBS containing 2000 SHG-44 cells was added to the Transwell chamber, and medium with 20% FBS was added to the wells of the 24-well plate. After culture for 48 h, cells that migrated to the bottom of the Transwell chamber were fixed at room temperature for 30 min with 4% paraformaldehyde and stained with a crystal violet solution. The stained cells were photographed and counted.

### Statistical analysis

Data were statistically analyzed using the R software (version 3.6.1). One-way analysis of variance (ANOVA) was used to explore the differences in the expression of *DSN1* in tumors and normal tissues. The chi-square test was used to explore the relationship between *DSN1* mRNA levels and clinical molecular characteristics. Univariate and multivariate regional models were used to explore the effects of clinical molecular characteristics on patient prognosis. An ROC curve was used to evaluate the prognostic value of the *DSN1* gene. Pearson correlation coefficient was used to analyze the expression relationship between *DSN1* and other genes in LGGs. Kaplan – Meier survival analysis was used to test the effect of *DSN1* or its methylation sites on the overall survival of patients. A p-value of less than 0.05 was considered statistically significant.

## Results

### The expression status of DSN1 in tumor tissues

**Figure 1.**
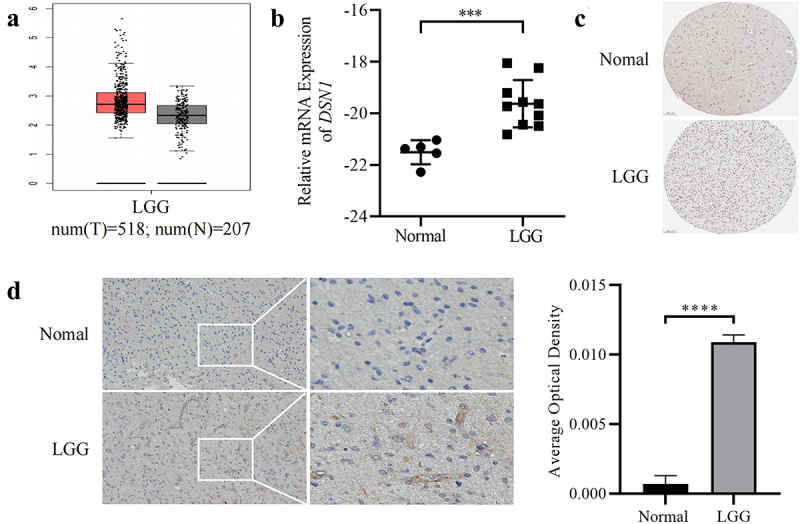
The expression of *DSN1* in LGG. (a) GEPIA showed that the expression level of *DSN1* in LGG (*n* = 518) was higher than that of normal brain tissue (*n* = 207). The method for differential analysis is one-way ANOVA, using disease state (tumor or normal) as variable for calculating differential expression. (b) mRNA levels of DSN1 in the laboratory samples of normal brain tissues and LGG tissues. (c) Immunohistochemical results in HPA, *DSN1* is different in lower-grade glioma and normal brain tissue. The pictures come from the open public platform and meet the use standards. (d) Immunohistochemical staining of *DSN1* in the LGG samples and normal brain tissues from laboratory. *** *p* < .001, **** *p* < .0001.

Many genes have disordered expression in LGG. Disordered genes, such as *PDCD1*, are related to the pathological progression of gliomas. Therefore, we used the Gene Expression Profiling Interactive Analysis (GEPIA) database to explore the differences in *DSN1* expression between LGG and normal brain tissues. According to the box diagram in the GEPIA database (Figure 1a), the mRNA expression level of *DSN1* in tumor tissues was significantly higher than that in normal control tissues. Besides, the clinical samples from the laboratory, including 5 cases of normal brain tissues and 10 cases of LGG samples, also showed the consistent results of mRNA levels of DSN1 in LGG (Figure 1b). Secondly, to understand the expression status of *DSN1* at the protein level, we used The Human Protein Atlas (HPA) database to explore differences in *DSN1* protein expression between LGGs and normal brain tissues. The results obtained from the HPA database showed that *DSN1* protein expression in LGG was markedly higher than that in normal tissues (Figure 1c). To further validate these results, laboratory brain samples were used to explore the protein levels of *DSN1* in LGG. Immunohistochemistry (IHC) results showed that the expression of *DSN1* was significantly elevated in LGG samples compared to normal brain tissues (Figure 1d). These results suggest that the high expression of *DSN1* in LGG may be involved in regulating the pathological process of LGG.

### The relationship between DSN1 and clinical features

**Figure 2.**
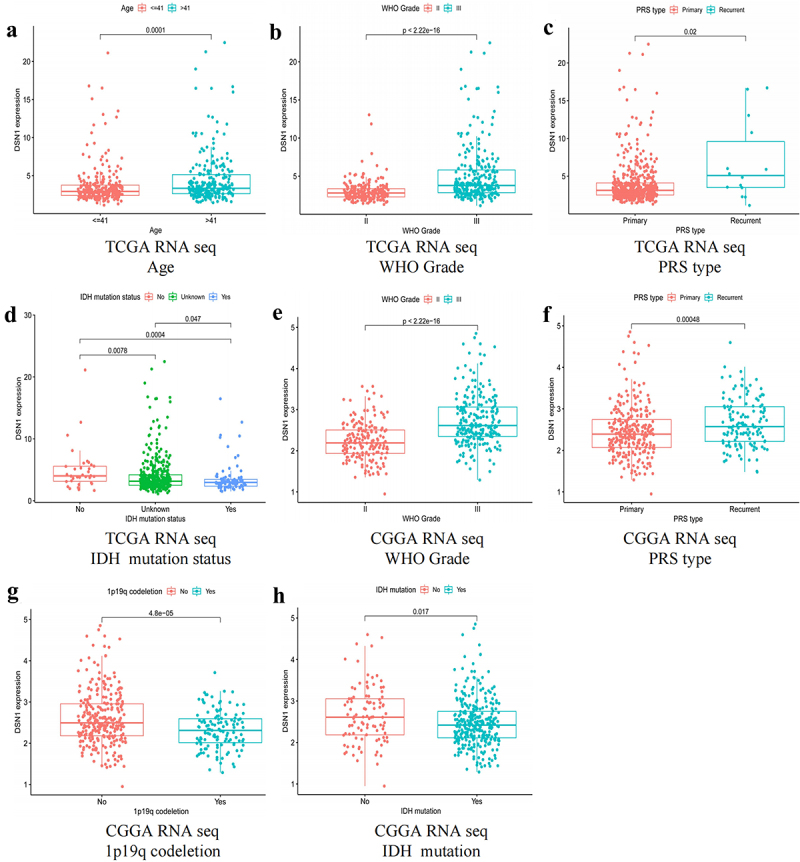
The relationship between *DSN1* expression level and various clinical factors in different databases. (a-d) *DSN1* expression in TCGA-RNA seq is related to age, WHO grade, PRS type and IDH mutation status. (e-h) *DSN1* expression in CGGA-RNA seq and WHO grade, PRS type, 1p19q codeletion and IDH mutation status. The chi-square test was used to explore the relationship between *DSN1* and clinical molecular characteristics.

To explore the relationship between *DSN1* mRNA and clinical molecular characteristics, we used the chi-square test to analyze TCGA-RNA-Seq and CGGA-RNA-Seq sequences. The results of the TCGA-RNA-seq database showed that in LGG, the expression level of *DSN1* was markedly increased in age > 41 vs. <41 years (*p* = .0001, Figure 2a), grade III vs. grade II (*p* = 2.22 × 10^−16^, Figure 2b), and recurrence vs. primary (*p* = .02, Figure 2c). However, in LGG patients with *IDH* mutations (p*=*0.0004, Figure 2d), the expression level of *DSN1* was found to be significantly reduced. To comprehensively explore the relationship between *DSN1* expression level and clinical characteristics, we used the CGGA dataset. In the CGGA-RNA-Seq dataset, it was also found that the expression level of *DSN1* was positively correlated with high WHO grade (*p* < 2.22e-16, Figure 2e) and poor PRS type (*p=*0.00048, Figure 2f), but the expression of *DSN1* was negatively correlated with a 1p19q codeletion (*p=*4.8e-05, Figure 2g) and mutation of *IDH* (*p=*0.017, Figure 2h). The results from the TCGA and CGGA datasets showed that the expression level of *DSN1* was related to poor clinical features. Therefore, the uncertainty of independent data was eliminated to a certain extent, and implies that *DSN1* may have a relationship with the prognosis of LGG.

### DSN1 is associated with LGG prognosis

**Figure 3.**
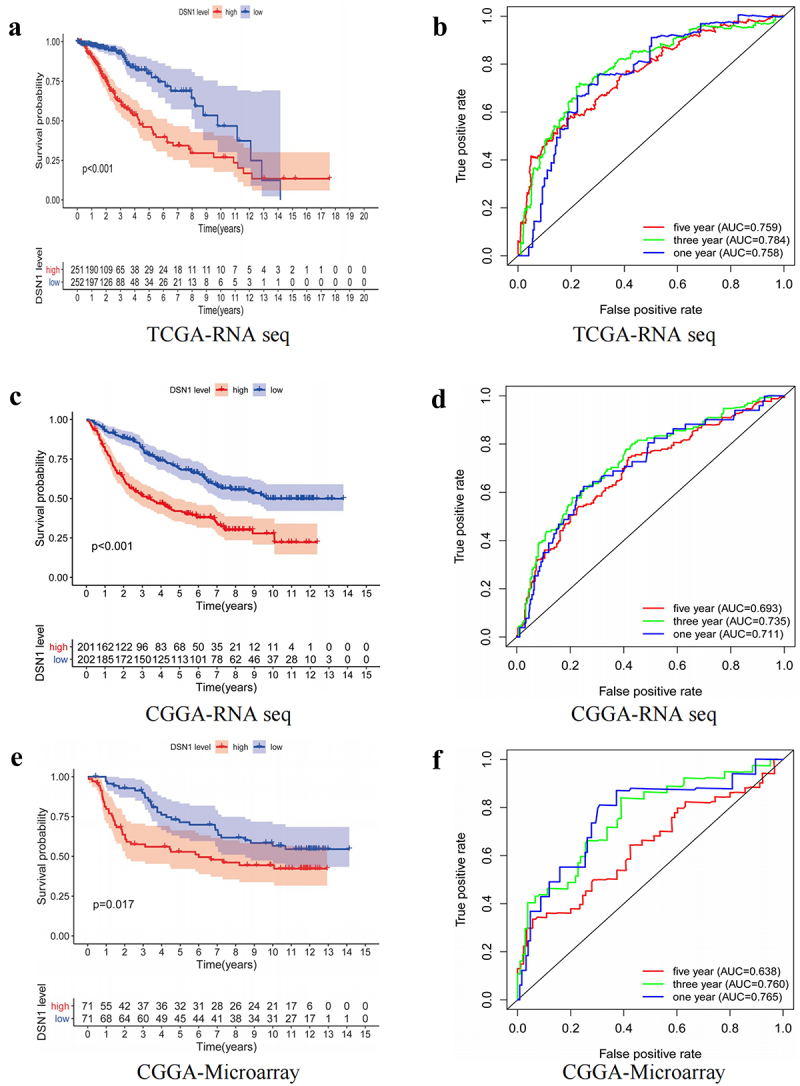
Survival curves and corresponding ROC curves in three different databases. (a, b) Survival curve and ROC curve in TCGA-RNA seq. In the TCGA-RNA seq database, the median survival rate of the *DSN1* high expression group was 1.751 years {p = .02952, 95% CI (0.7294-0.845)}, and the median survival rate of the *DSN1* low expression group was 2.1589 years {p = .01694, 95% CI (0.9139-0.980)}. (c, d) Survival curve and ROC curve in CGGA-RNA seq in the CCGA sequencing database, the median survival rate of the *DSN1* high expression group was 2.8712 years {p = .03562, 95% CI (0.463-0.603)}, while the *DSN1* low expression group had a median survival rate of 6.025 years {p = .03552, 95% CI (0.590-0.730)}. (e, f) Survival curve and ROC curve in CGGA-Microarray. Kaplan-Meier analysis was used to analyze the prognosis of LGG patients. In the CGGA-Microarray database, the median survival rate of the *DSN1* high expression group was 4.4329 years {p = .0607, 95% CI (0.438-0.677)}, while the *DSN1* low expression group had a median survival rate of 8.814 years {p = .0618, 95% CI (0.474-0.718)}.

We found that *DSN1* may be an oncogene associated with the prognosis of LGG. To further understand the impact of *DSN1* on the prognosis of patients with LGG, we used the Kaplan – Meier method to draw the overall survival (OS) curve. We found that the high-*DSN1* expression group had poor survival in the TCGA dataset (Figure 3a). In addition, it was also found that the high *DSN1* expression group had a shorter survival time in the CGGA-RNA-Seq and CGGA-microarray datasets (Figure 3c,e). This indicates that the *DSN1* expression level may affect the prognosis of patients with LGG.

**Figure 4.**
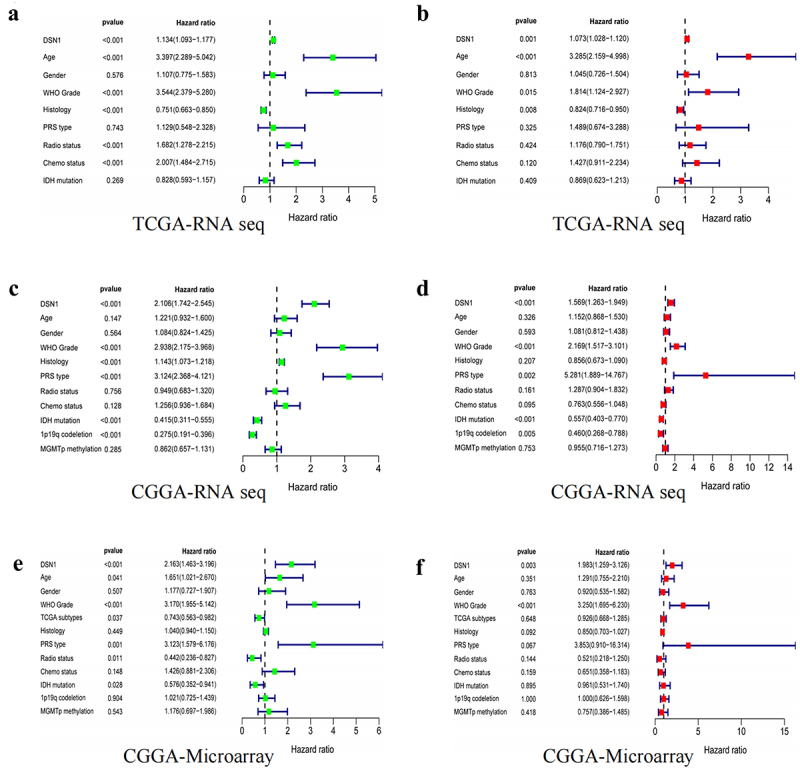
Single-factor and multi-factor analysis results in different databases. (a, b) Results in the TCGA-RNA seq database (c, d) results in the CGGA-RNA seq database (e, f) Results in the CGGA-Microarray database. Univariate and multivariate region models were used to explore the risk of clinical molecular characteristics on the prognosis of patients.

Therefore, we aimed to elucidate the relationship between *DSN1* expression and LGG prognosis. Subsequently, we used three different datasets to perform univariate and multivariate regression analyses (Figure 4a–f). The results showed that *DSN1* is a risk factor affecting the prognosis of patients with LGG. To verify the diagnostic value of *DSN1* for LGG prognosis, we constructed a corresponding Receiver Operating curve (ROC). The results of the ROC curve showed that in the three datasets, including the TCGA, CGGA-RNA-Seq, and CGGA-microarray datasets, the area under the curve (AUC) values for 1-year and 3-year patient survival were both greater than 0.7. The AUC for 5-year survival was also close to 0.7 (Figure 3b,d,f). This implies that *DSN1* can be used as a potential biomarker to evaluate the OS of patients and has diagnostic value.

### The methylation status of DSN1 in LGG

**Figure 5.**
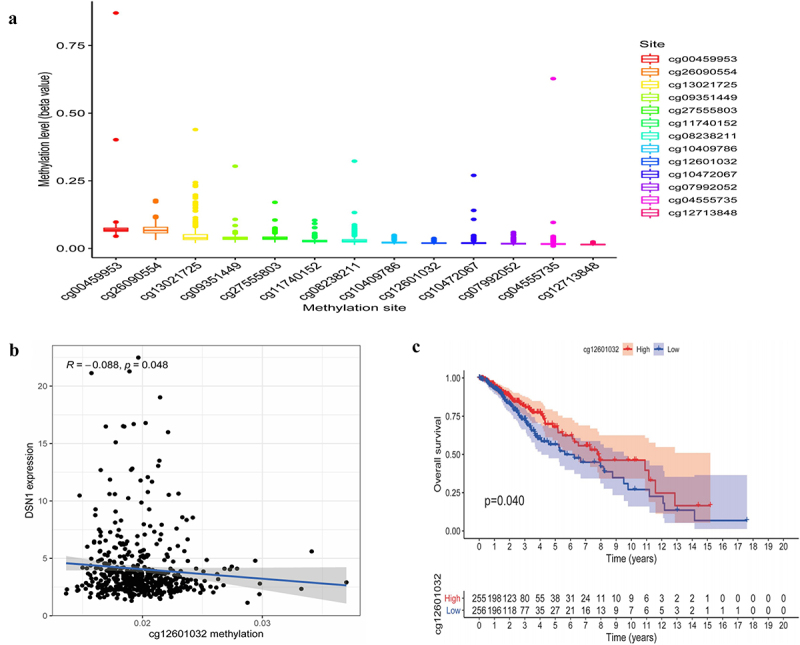
Methylation is involved in the regulation of *DSN1*. (a) Some methylation sites related to *DSN1*. (b) Methylation at cg12601032 is negatively correlated with *DSN1* expression. (c) Survival curve of patients with hypermethylation at cg12601032. The median survival rate of the hypermethylated group was 1.9425 years (*p* = .02308, 95%CI (0.8400-0.931)), and the median survival rate of the hypomethylated was 1.751 years (*p* = .02615, 95% CI (0.7977-0.900)).

To explore the regulatory effect of methylation status of *DSN1* in LGG, we analyzed 501 LGG methylation data points from the UCSC Xena database and obtained 13 *DSN1* CpG methylation sites (Figure 5a). Pearson’s correlation analysis showed that the methylation of cg12601032 was negatively correlated with *DSN1* expression (Figure 5b, *p=*0.048,*R* = −0.088). Finally, the Kaplan – Meier survival curve showed that patients in the hypermethylated cg12601032 group had better survival (Figure 5c). This provides further evidence that the hypermethylation of CpG sites reduces the expression of *DSN1*, which may affect the pathological progression of gliomas.

### DSN1 is associated with immune infiltration in patients with LGG

**Figure 6.**
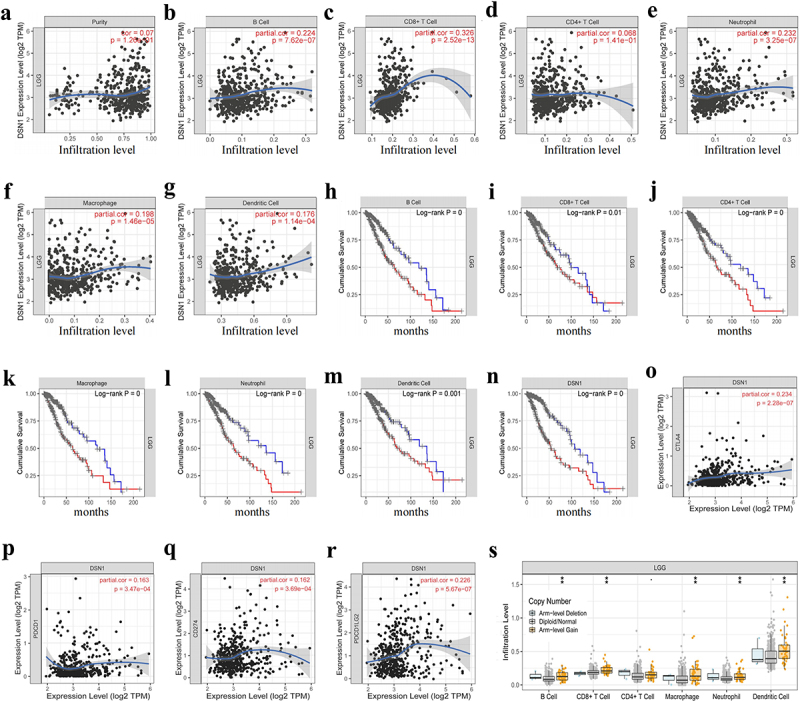
The relationship between *DSN1* and immune cells in LGG. (a-g) in LGG, *DSN1* is related to the abundance of B cell, CD8+ T cell, CD4+ T cell, macrophage, neutrophil, and dendritic cell. (h-n) the survival curve of different immune cell infiltration in LGG. (o-r) the relationship between *DSN1* expression and *PDCD1*, *CD274*, *PDCD1LG2*, *CTLA4*. (s) The relationship between somatic copy variation and the abundance of immune infiltration. The pictures are from the open online analysis platform, and the use of the pictures meets the standards. ** *p* < .01.

To reveal the relationship between *DSN1* and LGG immune cell infiltration, through the “gene” module in the Tumor Immune Estimation Resource (TIMER) library, we found that the infiltration of some immune cells in LGG was positively related to the expression of *DSN1* (Figure 6a–g). The OS curve results showed that among the six different immune cells, patients with LGG and high immune infiltration had a poor prognosis, and patients with LGG and high *DSN1* expression also had a poor prognosis (Figure 6h–n). This may be due to the involvement of immune-infiltrating cells in the regulation of LGG development. Third, the correlation module results showed that *DSN1* was related to some well-known tumor immune-related biomarkers, such as *PDCD1*, *CD274*, *PDCD1LG2*, and *CTLA4* (Figure 6o–r). Notably, *PD1* is related to tumor immune escape; therefore, we suspect that *DSN1* may be involved in this process. The results of the somatic module showed that arm-level gain affected the changes in the abundance of the six immune cell types in LGG (Figure 6s). In addition, we found that the number of infiltrated immune cells in the *IDH* mutation group was significantly lower than that in the*IDH* wild-type *IDH* group (Fig. S1). Based on the above experimental results, it is clear that *DSN1* has a regulatory effect on the LGG immune microenvironment; however, the detailed regulatory mechanism of *DSN1* needs to be confirmed in future studies.

### GSEA and co-expression analysis

**Figure 7.**
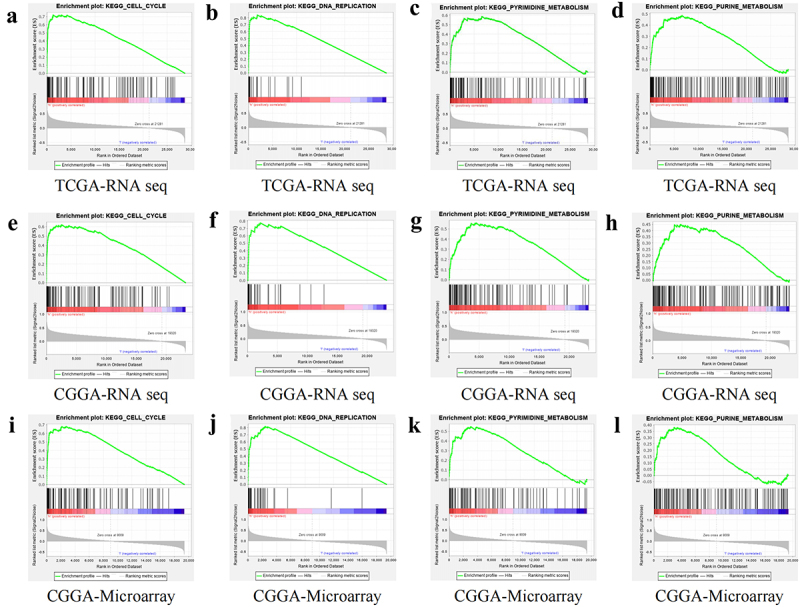
GSEA analysis based on three different databases. (a-d) GSEA result in TCGA-RNA seq (e-h) GSEA result in CGGA-RNA seq (i-l) GSEA result in CGGA-Microarray.

**Figure 8.**
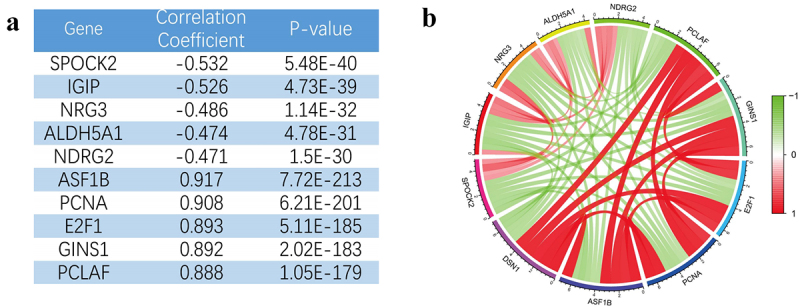
Co-expression analysis based on TCGA-RNA seq (a) Co-related gene name, *p* value and correlation coefficient. (b) Co-expression relationship diagram related to *DSN1*. Pearson correlation coefficient analysis was used to analyze the relationship between *DSN1* and other genes.

GSEA revealed potential biological functions of *DSN1*. The GSEA results from TCGA dataset showed that Kyoto Encyclopedia of Genes and Genomes (KEGG) cell cycle, DNA replication, pyrimidine metabolism, and purine metabolism were highly related to *DSN1* (Figure 7a–d). This suggests that *DSN1* may be involved in tumor proliferation. Subsequently, we performed GSEA on the CGGA chip and sequencing data. Surprisingly, we found that the results from the CGGA were similar to those from TCGA (Figure 7e–l). This ruled out the uncertainty of TCGA results to a certain extent. The results of the three datasets consistently suggested that *DSN1* may participate in the pathological progression of LGG through various biological pathways (Tables S2–4). In addition, the results of co-expression analysis showed that *DSN1* was positively correlated with *ASF1B*, *PCNA*, *E2F1*, *GINS1*, and *PCLAF*. Furthermore, *DSN1* expression negatively correlated with *SPOCK2*, *IGIP*, *NRG3*, *ALDH5A1*, and *NDRG2* expression (Figure 8a). We also constructed a co-expression diagram (Figure 8b). Through this search, we found that overexpression of the positively correlated gene, *ASF1B*, accelerated the proliferation of cervical cancer cells. Similarly, it has been reported that *PCNA* is involved in the proliferation and metastasis of glioma cells. The results of the co-expression and GSEA indicated that *DSN1* is highly correlated with tumor proliferation, which further confirms the credibility of our research. Moreover, the functions of these synergistic genes (Figure 8a) revealed the role of *DSN1*.

### Meta-analysis

**Figure 9.**
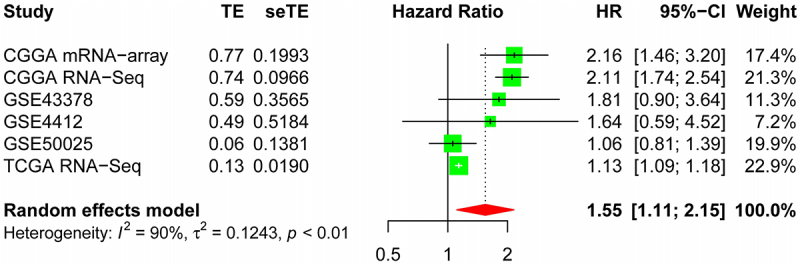
Meta-analysis on the prognosis of *DSN1* and LGG. Including the names of 6 data sets, HR values, 95% confidence intervals and weights. According to I^2^ greater than 50%, we choose the random effects model.

Meta-analysis was performed to further understand the role of *DSN1* in the prognosis of LGG using different datasets. The results in these six datasets show that the expression level of *DSN1* was closely related to the survival of patients with LGG: TCGA RNA-Seq (HR = 1.13,95%CI [1.09;1.18]), CGGA-mRNA-array (HR = 2.16, 95%CI [1.46;3.20]), CGGA-RNA-Seq (HR = 2.11, 95%CI [1.74;2.54]), GSE43378 (HR = 1.81, 95%CI [0.90;3.64]), GSE4412 (HR = 1.64, 95%CI [0.59;4.52]), GSE50025 (HR = 1.06, 95%CI [0.81;1.39]) (Figure 9). These results imply that *DSN1* could be used as a potential biomarker to evaluate the prognosis of patients with LGG.

### Knockdown of DSN1 inhibits the proliferation and invasion of LGG cells

**Figure 10.**
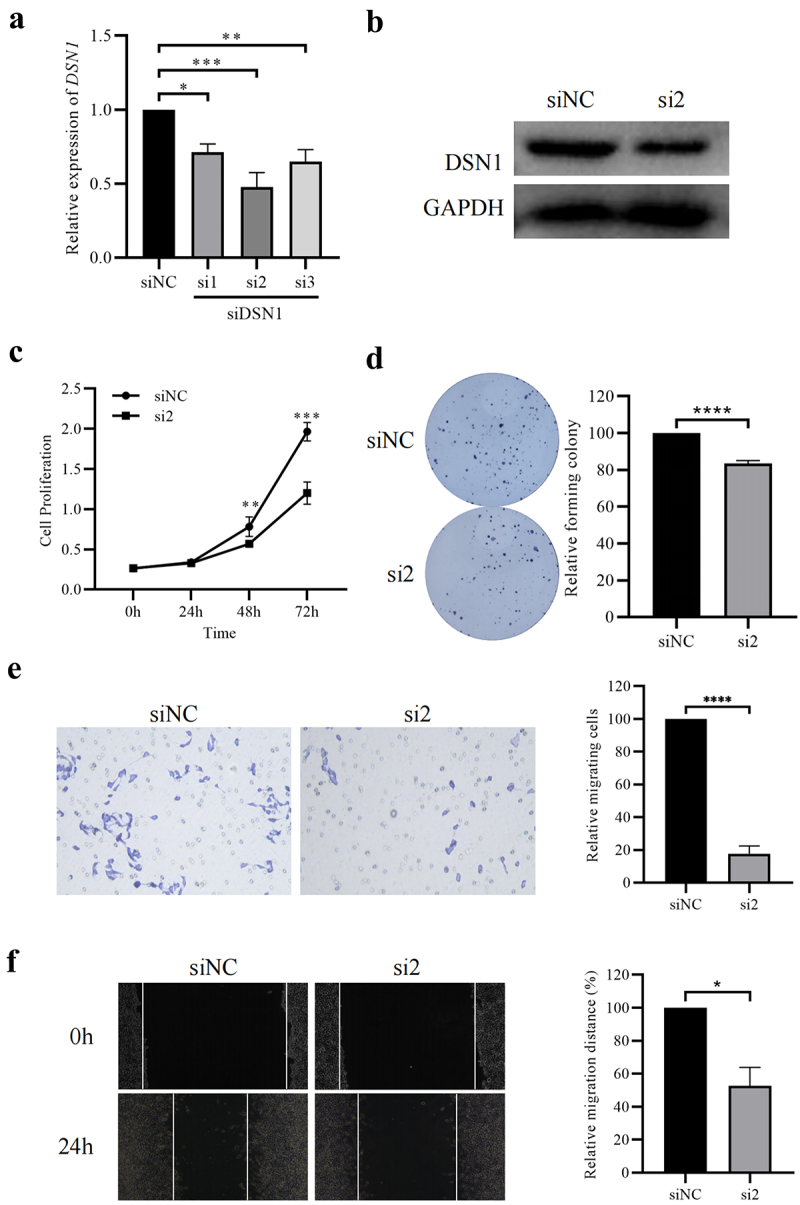
Knockdown of *DSN1* significantly inhibited the proliferation and invasion of LGG cells. (a) Knockdown efficiency of siRNAs targeting on *DSN1* in SHG-44 cells. (b) Knockdown result of *DSN1* treated with si2 at protein level. (c) CCK8 results of siRNA treated cells at different time points. (d) The results of colony formation of SHG-44 cells treated with different siRnas. (e) The relative distances of SHG-44 cells in wound healing assay. (f) The staining results of invading cells treated with siRNA in Transwell assay. * *p* < .05, ** *p* < .01, *** *p* < .001, **** *p* < .0001.

To further validate the scientific reliability of this study, *in vitro* experiments were performed to validate the effect of *DSN1* on LGG cells. First, the knockout efficiency of siRNAs targeting *DSN1* was identified, and treatment with si2 of the three siRNAs showed the most significant reduction in *DSN1* expression, by approximately half (Figure 10a); knockdown results at protein level was also presented (Figure 10b). Therefore, si2 was used in subsequent experiments. Second, cell-counting-kit-8 (CCK8) assays confirmed that SHG-44 cells with *DSN1* knockdown showed significantly lower cell proliferation at 48 h and 72 h compared than control cells (Figure 10c). The colony formation assay confirmed that the ability of siRNA-treated cells to form colonies was significantly impaired (Figure 10d). These results suggested that *DSN1* significantly promoted the proliferation of LGG cells. Furthermore, the invasiveness of SHG-44 cells was significantly reduced by knockdown of *DSN1* expression in both wound healing and Transwell assays (Figure 10e,f). Collectively, these results further confirm the role of *DSN1* in the malignant progression of LGG, and provide an important contribution to the detailed pathogenesis of LGG.

## Discussion

LGGs are common malignant brain tumors with great inherent heterogeneity. Traditional comprehensive treatment methods have greatly improved patient survival rates. However, owing to the invasive growth and rapid metastasis of gliomas, many patients experience recurrence and drug resistance. Moreover, 70% of LGGs progress to more malignant gliomas within ten years, which seriously endangers the lives of patients.^33^ Advances in sequencing technologies have promoted the prognostic diagnosis of LGG. To the best of our knowledge, this is the first study to reveal the biological functions of *DSN1* using a variety of public databases. It also demonstrates the influence of the disorder of *DSN1* expression on the prognosis of patients and provides a powerful tool for improving the prognosis of patients with LGG.

First, we explored the expression status of *DSN1* in LGG. Immunohistochemical staining of the HPA database and laboratory results consistently showed that the expression of *DSN1* was markedly higher in LGG than in normal brain tissue. Related reports have revealed that *DSN1*, a carcinogen, is highly expressed and correlated with the proliferation of colorectal adenomas. Moreover, the increase in *DSN1* expression is related to the progression of *HER2* heterogeneous breast cancers.^34^ These studies show that *DSN1* is involved in the progression of a variety of malignant tumors and plays an important regulatory role in the malignant progression of tumors. Therefore, we speculated that the abnormally high expression of *DSN1* may also be related to the pathological development of LGG.

To further clarify the relationship between *DSN1* expression levels and the clinical characteristics of LGG, we performed Wilcoxon and Kruskal – Wallis rank sum tests to analyze the profiles in TCGA and CGGA databases. Older age, high WHO classification, and poor PRS type were positively correlated with *DSN1* expression. The presence of 1p19q co-deletion and *IDH* mutations was negatively correlated with the expression of *DSN1*. Studies have reported that age is an important predictor of glioma survival,^35^ and the prognosis of patients with high WHO grade gliomas is poor.^36^ Patients with gliomas, with 1p19q codeletion and *IDH* mutations, tend to have a relatively good prognosis.^37,38^ Similar to most biological targets of LGG, the expression level of *DSN1* is closely related to the malignant features of tumors. Thus, *DSN1* may be associated with cancer progression.

To evaluate the role of *DSN1* in prognosis, survival curves showed that the overall survival of patients in the *DSN1* high-expression group was shorter than that of patients in the low-expression group. Subsequently, the ROC curve indicated that *DSN1* had a good prognostic value for patients with LGG. Multivariate and meta-analyses suggest that *DSN1* is a risk factor affecting the prognosis of LGG. Consistent with previous studies, *DSN1* has been shown to be a risk factor for liver cancer.^39^ It has also been reported that abnormally high expression of *DSN1* can promote the proliferation of colorectal cells and reduce the overall survival rate of patients.^40^

We discovered that the expression level of *DSN1* was negatively correlated with cg12601032 methylation sites and that the prognosis of patients with LGG in the hypomethylation group was poor. Similar to other studies, DNA methylation is considered an important factor in the prediction of LGG prognosis. It has been reported that the methylation level of the MGMT promoter can be regarded as a predictor of the risk of hypermutation during LGG recurrence and benefit the clinical management of patients. Similarly, *SHOX2* expression or methylation has been shown to be a prognostic indicator of the survival of patients with LGG.^41,42^ Collectively, these results suggested that abnormally high expression and methylation of *DSN1* are potent independent predictors of poor prognosis in patients with LGG.

In addition to the unlimited proliferative ability of tumor cells due to their heterogeneity of tumor cells, infiltrating immune cells in the tumor microenvironment cannot recognize and attack heterogeneous tumor cells in the pathological process of LGG, which promotes the poor prognosis of LGG patients.^43^ Some studies have shown that the interaction and regulation between infiltrating immune cells and tumor cells form a special immunosuppressive microenvironment of LGG, which promotes the malignant progression of tumor cells; however, the detailed mechanism remains to be elucidated.^44,45^ Therefore, this study aimed to explore whether *DSN1*, a tumorigenic molecule, has a regulatory effect on the formation of the LGG tumor immune microenvironment. First, in the “Gene module” of TIMER, *DSN1* had a positive expression relationship with a variety of infiltrating immune cells (e.g. CD8+T cells and macrophages). The infiltration levels of both CD8+T cells and macrophages in the pathological process of LGG are negatively correlated with the clinical prognosis of patients with glioma.^46,47^ More importantly, the expression of *DSN1* negatively correlated with *IDH* mutations, and the infiltration level of immune cells in the *IDH* mutation group was substantially lower than that in the wild-type group. This is consistent with previous reports that the wild-type group with *IDH* has a high immune cell score and is related to the poor prognosis of LGG.^48^ The results of this study also suggest that an increased number of infiltrating immune cells can contribute to the poor prognosis of patients (Figure 6h–n). It can be inferred from the above evidence that *DSN1* has a regulatory effect on the immune microenvironment of LGG and affects the prognosis of patients; however, more detailed regulatory mechanisms need to be confirmed by more studies. More importantly, there was a positive relationship between the mRNA expression of *DSN1* and the expression of many well-known immune checkpoints (e.g., PD1 and PDL1). Increased PD1/PDL1 expression in tumor tissues promotes the immune escape of tumor cells.^49,50^ Therefore, new anti-tumor immunotherapies based on PD-1/PD-L1 immunosuppressants have achieved good clinical results in melanoma tumor.^51^ However, the efficacy of PD1/PDL1 inhibitors in glioma clinical trials is considerably lower than that in other tumors, and they have substantial toxicity and side effects.^52^ The bottleneck of this treatment is mainly due to the limited expression and regulatory mechanism of PDL1 in gliomas. Therefore, revealing the regulatory relationship between *DSN1* and PD1/PDL1 will help clarify the complex network of immune regulation in LGG, improve our understanding of the molecular regulatory mechanism of *DSN1* in LGG, and explore the potential value of *DSN1* as an anti-tumor combined immunotherapy.

To determine the potential mechanism, co-expression and GSEA were used to further reveal the biological functions of *DSN1*. We found that *DSN1* positively correlated with genes such as *PCNA* and *ASF1B*. Interestingly, studies have found that the upregulation of *PCNA* promotes the malignant proliferation of glioma cells. It has also been confirmed that the upregulation of *PCNA* expression leads to a poor prognosis in patients with osteosarcoma.^53,54^ In addition, studies have reported that increased *ASF1B* expression can promote the malignant proliferation of a variety of tumor cells, such as renal cell carcinoma, breast, prostate, and cervical cancer.^55–57^ There is a good chance that the synergy between *DSN1* and the co-expressed genes contributes to LGG development.

The GSEA results of the three datasets consistently showed that cell cycle, DNA replication, pyrimidine metabolism, and purine metabolism were substantially enriched in the high*-DSN1* expression group. Previous studies have shown that *DSN1* is related to the cell cycle and is involved in osteosarcoma cells.^22^ In addition, it has been established that cell cycle pathway-related genes promote the proliferation of liver and gastric cancer.^58,59^ Therefore, we speculate that *DSN1* may promote the malignant proliferation of LGG cells by regulating the cell cycle pathway, and abnormally high expression of *DSN1* leads to an unsatisfactory prognosis for patients with glioma. To the best of our knowledge, this is the first study to use multiple databases to explore the relationship between *DSN1* expression and LGG prognosis. Furthermore, laboratory results identified a key role of *DSN1* in regulating proliferation and invasion during LGG pathological progression. In conclusion, *DSN1* is an oncogene that explicitly contributes to the malignant progression of LGG and can be recognized as a potential biomarker for the prognostic diagnosis of LGG.

Our study had some limitations. We chose three different public databases for retrospective research, therefore, there was a lack of complete clinical data on the surgical methods, types, and dosages of radiotherapy and chemotherapy. Second, multiple databases were involved, therefore there may be some differences in the treatment of patients. However, the three databases selected had large sample sizes of up to 1046 cases involving multiple countries and races. Moreover, public databases are open and allow researchers from all over the world to participate in related studies, which greatly reduces research costs and time. Therefore, we believe that the advantages of public databases outweigh their limitations. Finally, the research is based only on the reanalysis of public data, therefore, it is only a potential possibility and index that needs more research for further verification.

This study revealed, the adverse effect of *DSN1* on the prognosis of patients with LGG, and that high expression of *DSN1* is negatively regulated by its methylation site. In addition, *DSN1* mRNA and cg12601032 are potential targets for the diagnosis and treatment of LGG. In addition, this study partially revealed the regulatory mechanism of *DSN1* in the pathological process of LGG through analysis of biological information. The laboratory results confirmed that *DSN1* markedly promoted the proliferative and invasive capacity of LGG cells. Ultimately, the genetic level can contribute to the pathogenesis of LGG and expand the molecular understanding of *DSN1* in tumors.

## Supplementary Material

Supplementary file.docx

## Data Availability

The original data in the manuscript can be obtained by contacting the corresponding author.

## Abbreviations

LGG: lower-grade glioma
GBM: glioblastoma multiforme
DSN1: Dosage suppressor of Nnf1
TCGA: The Cancer Genome Atlas Program
CGGA: Chinese Glioma Genome Atlas
GEO: Gene Expression Omnibus
GEPIA: Gene Expression Profiling Interactive Analysis
GSEA: Gene set enrichment analysis
