# Supplementary material for: DSN1 may predict poor prognosis of lower-grade glioma patients and be a potential target for immunotherapy
Source: Cancer Biol Ther. 2024 Nov 18;25(1):2425134. doi: 10.1080/15384047.2024.2425134 (PMC11581156; doi:10.1080/15384047.2024.2425134)
Supplement: Supplementary file.docx [file KCBT_A_2425134_SM1214.docx]

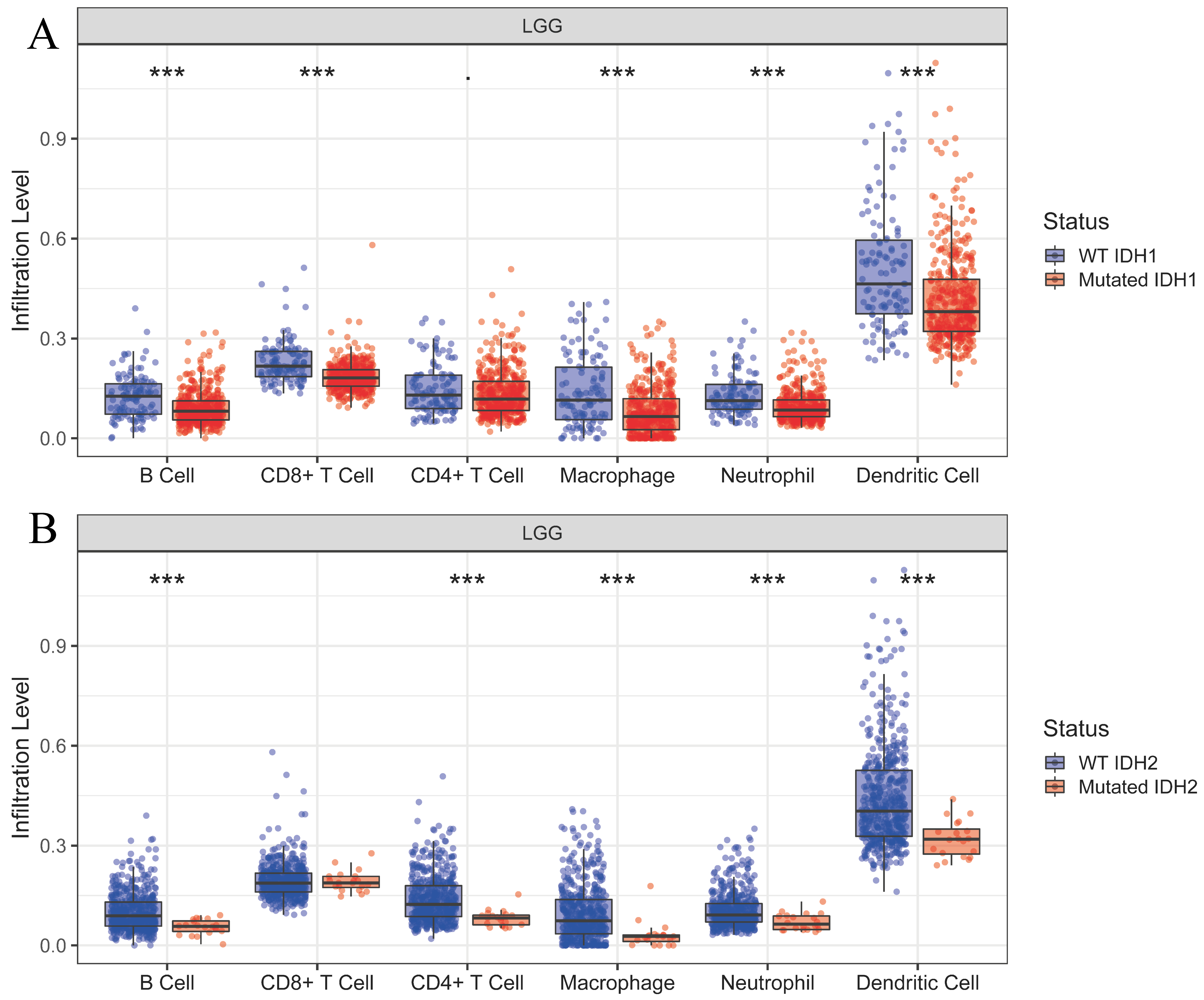


**Fig S1:** Based on the mutation module of the TIMER platform, compare the immune penetration levels of different IDH states in LGG. A: Comparison of the level of immune infiltration between IDH1 wild type (WT IDH1) (118 cases, 22.1%) and IDH1 mutant type (IDH1 mutated) (415 cases, 77.9%). B: Comparison of the level of immune infiltration between IDH2 wild-type (WT IDH1) (96.2%) and IDH2 mutant type (IDH1 mutated) (3.8%).

**TABLE S1 Clinical information of 503 LGG patients in the TCGA database.**

| **Covariates** | **Type** | **Total** | **Percentages (%)** |
| --- | --- | --- | --- |
| Age | <=41 | 260 | 51.69% |
| Age | >41 | 243 | 48.31% |
| Gender | Female | 225 | 44.73% |
| Gender | Male | 278 | 55.27% |
| WHO Grade | II | 243 | 48.31% |
| WHO Grade | III | 260 | 51.69% |
| Radio status | No | 187 | 37.18% |
| Radio status | Unknown | 72 | 14.31% |
| Radio status | Yes | 244 | 48.51% |
| Chemo status | No | 167 | 33.20% |
| Chemo status | Unknown | 66 | 13.12% |
| Chemo status | Yes | 270 | 53.68% |
| Histology | A | 64 | 12.72% |
| Histology | AA | 127 | 25.25% |
| Histology | MG | 127 | 25.25% |
| Histology | O | 107 | 21.27% |
| Histology | OA | 78 | 15.51% |
| PRS type | Primary | 489 | 97.22% |
| PRS type | Recurrent | 14 | 2.78% |
| IDH mutation status | No | 34 | 6.76% |
| IDH mutation status | Unknown | 378 | 75.15% |
| IDH mutation status | Yes | 91 | 18.09% |
| expression | High | 251 | 49.90% |
| expression | Low | 252 | 50.10% |
| methylation | High | 251 | 49.90% |
| methylation | Low | 252 | 50.10% |

**TABLE S2** **The gene set enriches the high *DSN1* in TGGA RNA-seq database**

| **Name** | **NES** | **NOM p-val** | **FDR q-val** |
| --- | --- | --- | --- |
| KEGG CELL CYCLE | 2.269 | 0 | 0 |
| KEGG DNA REPLICATION | 2.006 | 0 | 0.0248 |
| KEGG PYRIMIDINE METABOLISM | 1.955 | 0 | 0.0217 |
| KEGG PURINE METABOLISM | 1.963 | 0.002 | 0.0187 |

NES: normalized enrichment score; NOM: nominal; FDR: false discovery rate. Gene sets with NOM p-value <0.05 and FDR q-value <0.25 were considered as significantly enriched.

**TABLE S3** **The gene set enriches the high *DSN1* in CGGA Microarray**

| **Name** | **NES** | **NOM p-val** | **FDR q-val** |
| --- | --- | --- | --- |
| KEGG CELL CYCLE | 2.175 | 0 | 9.02E-04 |
| KEGG DNA REPLICATION | 1.919 | 0 | 0.026 |
| KEGG PYRIMIDINE METABOLISM | 1.812 | 0 | 0.053 |
| KEGG PURINE METABOLISM | 1.674 | 0.002 | 0.140 |

NES: normalized enrichment score; NOM: nominal; FDR: false discovery rate. Gene sets with NOM p-value <0.05 and FDR q-value <0.25 were considered as significantly enriched.

**TABLE S4** **The gene set enriches the high *DSN1* in CGGA RNA-seq database**

| **Name** | **NES** | **NOM p-val** | **FDR q-val** |
| --- | --- | --- | --- |
| KEGG CELL CYCLE | 1.822 | 0.012 | 0.1096 |
| KEGG DNA REPLICATION | 1.976 | 0 | 0.0729 |
| KEGG PYRIMIDINE METABOLISM | 1.807 | 0.001 | 0.096 |
| KEGG PURINE METABOLISM | 1.671 | 0 | 0.220 |

NES: normalized enrichment score; NOM: nominal; FDR: false discovery rate. Gene sets with NOM p-value <0.05 and FDR q-value <0.25 were considered as significantly enriched.
